# Electrochemical Flow-ELISA for Rapid and Sensitive Determination of Microcystin-LR Using Automated Sequential Injection System

**DOI:** 10.3390/s17071639

**Published:** 2017-07-16

**Authors:** Lesedi Lebogang, Jongjit Jantra, Martin Hedström, Bo Mattiasson

**Affiliations:** 1 Department of Biotechnology, Lund University, Box 124, SE-22100 Lund, Sweden; jongjit.jantra@gmail.com (J.J.); Martin.Hedstrom@biotek.lu.se (M.H.); Bo.Mattiasson@biotek.lu.se (B.M.); 2 Department of Biological Sciences and Biotechnology, Botswana International University of Science and Technology, Private Bag 16, Plot 10071, Palapye, Botswana; 3 CapSenze Biosystems AB, Scheelevägen 22, SE-22363 Lund, Sweden

**Keywords:** microcystin-LR, flow-ELISA, amperometric biosensor, sequential injection, detection

## Abstract

An amperometric immunoanalysis system based on monoclonal antibodies immobilized on Sepharose beads and packed into a micro-immunocolumn was developed for the quantification of microcystin-LR. Microcystin-LR (MCLR) was used as a reference microcystin variant. Inside the immunocolumn, free microcystins and microcystin-horseradish peroxidase (tracer) were sequentially captured by the immobilized antibodies, and the detection was performed electrochemically using Super AquaBlue ELISA substrate 2,2′-azinobis(3-ethylbenzothiazoline-sulfonic acid) (ABTS). The ABTS^●+^ generated by enzymatic oxidation of ABTS was electrochemically determined at a carbon working electrode by applying a reduction potential set at 0.4 V versus Ag/AgCl reference electrode. The peak current intensity was inversely proportional to the amount of analyte bound to the immunocolumn. The amperometric flow-ELISA system, which was automatically controlled through the CapSenze^TM^ (Lund, Sweden) computer software, enabled determination of MCLR as low as 0.01 µg/L. The assay time was very short (20 min for one assay cycle). In addition, the electrochemical signals were not significantly affected by possible interferences which could be present in the real samples. Along with the simplicity of automation, this makes the developed method a promising tool for use in water quality assessment.

## 1. Introduction

The global increase of potentially harmful cyanotoxins in surface water systems is instrumental to the search for simple, rapid and field-applicable analytical techniques for extensive freshwater monitoring [1,2]. Microcystins (MCs) are a group of hepatotoxic cyanotoxins with more than 80 variants commonly present in watercourses [3,4,5,6]. MCs have a common cyclic structure built of seven amino acids including the unique amino acid called 3-amino-9-methoxy-2,6,8-trymethyl-10-phenyldeca-4,6-dienoic acid (Adda) to which their toxicity is attributed to [7,8,9,10], and are responsible for a vast amount of animal and human poisonings and deaths around the world [11,12].

Health risks posed by these MCs have intensified the search for convenient and rapid techniques for their detection and removal [13]. Hence, methods related to MC detection have continued to be developed over the years. Batch analysis, e.g., conventional enzyme-linked immunosorbent assays (ELISA) have rapidly gained commercial interest, and are presently widely used due to their robustness and reliable results. Currently, microtitre-based ELISA kits that only target a few MC variants [9,13] dominate the commercial market. A generic MC immunoassay based on monoclonal antibodies (mAbs; AD4G2) that could recognize Adda and its derivatives with high sensitivity has been developed [13,14,15]. Nevertheless, long analysis times [16,17] and the non-reusability of the well-format ELISA make routine analysis of MCs costly [18]. Convenient and easy-to-use dipstick assays are also available for MC detection [13,19,20]. However, dipsticks only give a yes/no response, which may not be adequate where detailed information about the levels present is required [18]. For instance, the WHO guideline for drinking water of 1 µg/L MCLR equivalents dictates that the target sensitivity of detection techniques should be at least below this value [1,21]. Designs of automated flow immunoassay platforms interfaced with transduction systems to make continuous flow-analysis would therefore greatly simplify MCs analysis in terms of speed and sensitivity.

Flow-ELISA systems for the detection of various targets have previously been reported [16,17,22,23,24] and recently sequential flow-injection systems have gained popularity [25,26]. Notably, these systems have not by far been explored for MC detection as compared to biosensor systems where the working electrode surface serves both as the support material for biomolecule immobilization and acts as the immunoreactor. Conversely, the flow-ELISA set-up separates the reaction environment from the detection mode, in that way minimizing interferences [27]. Moreover, commercial MC ELISA kits are exclusively based on colorimetry, whereas flow-ELISA configurations offer the flexibility of interfacing different modes of detection such as colorimetric, electrochemical as well as photometric [18,26,28], and more importantly, transductions that can give real-time analysis, for example amperometric detection. The flow-ELISA with sequential injection mode combines the selectivity of the antibodies for target recognition with the direct transduction of the rate of the biocatalytic reaction into a current signal, thereby allowing a rapid, simple and direct determination while allowing sequential injections of small volumes of reagents and samples through automation [18,25].

The present work describes a sequential micro flow-ELISA based on amperometric detection using a fully automated and computer-controlled system named VersAFlo. This sequential injection analysis (SIA) system was developed in-house and its operations are described fully by Kumar et al. [26] and its system integration by Erlandsson et al. [29]. The amperometric VersAFlo-ELISA system offers a significantly reduced assay time, real-time information related to the assay as well as high reusability of the immunocolumn. A further advantage of the system is the visual reaction product in the immunocolumn before it enters into the amperometric detection chamber. The micro-ELISA immunocolumn was prepared by immobilizing Adda-specific antibodies on CNBr-activated Sepharose beads. Electrochemical detection was performed amperometrically using horseradish peroxidase (HRP) label and ABTS, a substrate that is rapidly oxidized by HRP to generate ABTS^●+^ [30,31].

## 2. Materials and Methods

### 2.1. Materials and Reagents

CNBr-activated Sepharose was purchased from GE Healthcare (Uppsala, Sweden). Super AquaBlue was purchased from eBioscience (San Diego, CA, USA), MCLR standard was from Enzo Life Sciences (Malmö, Sweden), Adda-specific monoclonal antibodies were from Abraxis LLC (Warminster, PA, USA). Float-A-lyzer G2 dialysis membrane (MWCO, 20 kDa) and HRP were purchased from Sigma-Aldrich Chemie GmbH (Steinheim, Germany). DS 110 screen-printed electrodes (SPEs) were from DropSens (Llanera, Spain). All other chemicals used in this work were of analytical grade. Buffers were prepared using deionized water purified with Millipore (18.2 MΩ cm resistivity) (Millipore, Bedford, MA, USA).

### 2.2. Periodate-Conjugation of Microcystin-Horseradish Peroxidase

Microcystin-HRP conjugation in this work was carried out following the method of Wilson and Nakane [32] with some modifications. Prior to the conjugation step, 2.5 mg/mL HRP was activated by oxidizing with freshly prepared 5 mM sodium periodate (NaIO_4_) in sodium acetate buffer (50 mM, pH 4.4) for 30 min. The reaction was carried out in total darkness at 4 °C. Glycerol (0.2% v/v) was used to stop the oxidation reaction. The reaction yielded aldehyde groups from the oxidized carbohydrate part of the HRP. The mixture was put in a 20 kDa dialysis membrane chamber (Float-A-lyzer G2, Rancho Dominguez, CA, USA) and dialyzed overnight against the same acetate buffer at 4 °C. Activated HRP (HRP-aldehyde) was covalently conjugated to 2 µM MCLR (1:1 v/v) by reacting for 2 h at room temperature. Then 4 mg/mL sodium borohydride (NaBH_4_) was used to reduce the Schiff bases formed between the aldehydes and amino groups of the MCLR at 4 °C for 1 h. The MCLR-HRP conjugate was purified by gel filtration chromatography using a Sephadex G25 column and the absorbance was measured on UV/Vis spectrometer at 403 and 238 nm for HRP and MCLR absorbance maxima, respectively. Fractions with high absorbance at their respective wavelengths were pooled. After the gel filtration step, the volume of the preparation was reduced using spin column filters (10 kDa cut-off). Before storage at −20 °C, 50% glycerol was added to the preparation to prevent freezing.

### 2.3. Antibody Immobilization on CNBr-Activated Sepharose Beads

The immobilization of Adda-specific monoclonal antibodies onto Sepharose beads surface was performed following the protocol by Kumar et al. [26]. CNBr-activated Sepharose 4B (0.25 g) was reconditioned by passing 100 mL of 1 mM HCl through a collection of beads placed on a sintered filter. Next, the gel was washed in 50 mL coupling buffer (10 mM phosphate buffer containing 0.5 M NaCl; pH 8.1). The buffer was thereafter filtered off, leaving about 5 mL of the gel. The gel was transferred into a tube and allowed to settle. The supernatant was removed and the remaining gel collected and kept at 4 °C until use. Antibodies (0.5 mg/L) were dissolved in PBS (100 mM pH 7.4) and mixed with the gel (1:1 v/v). The mixture was reacted overnight at 4 °C on a rocking table. The mixture was allowed to settle before removing the supernatant (kept for protein determination). The gel was washed twice with coupling buffer and the wash-fractions were kept as well. To block the remaining active groups, the gel was treated with 0.1 M Tris-HCl, pH 8.0 for 2 h at room temperature. Subsequent to the blocking step, the gel was washed with 3 alternating cycles of 0.1 M acetate buffer with 0.5 M NaCl; pH 4.0 and 0.1 M Tris-HCl, 0.5 M NaCl pH 8.0. For preservation, 0.02% (w/v) NaN_3_ was added to the preparation and kept at 4 °C until use. The Sepharose-antibody suspension is relatively stable when stored below 8 °C. Once the suspension is prepared it is readily available to replace the used column, making the process very convenient since it avoids preparation of fresh reagents every time. To prepare the immunocolumn, 15 µL of the suspension was loaded into a microcolumn using a low flow rate of phosphate buffer to avoid the formation of bubbles in the packed gels.

### 2.4. Amperometric Measurements by VersAFlo Sequential Injection System

**Figure 1:**
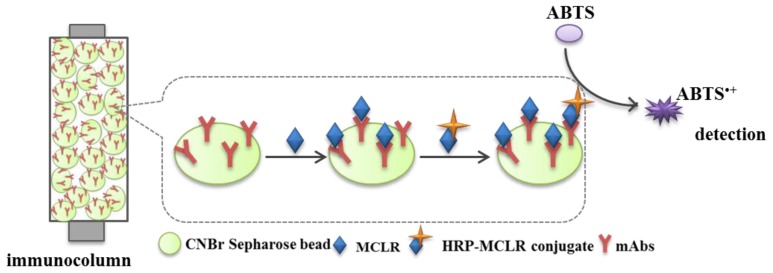
A schematic demonstration of the reaction taking place in the immunocolumn.

The flow-ELISA assay used in this work was a microscale technique that uses an enzyme-labelled analyte (MCLR-HRP) to compete for binding with a native analyte (MCLR) to the immobilized antibodies in the immunocolumn. In the reaction column a sequential binding competition [26] taking place as exemplified in Figure 1. The free MCLR is first injected, and right after the peroxidase-labelled MCLR is introduced.

**Figure 2:**
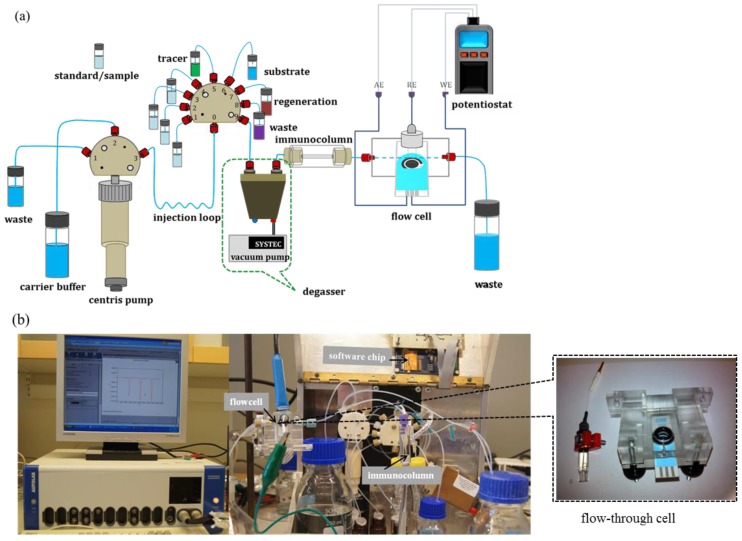
(**a**) Schematic representation of an automated sequential injection configuration (modified from Erlandsson et al. [29] and (**b**) Photograph of the experimental set-up with *insert* of a flow-through cell.

In the automatic sequential injection system named VersAFlo [26] (Figure 2), the reagents were passed in an arranged sequence to the reaction immunocolumn with the aid of a syringe pump and injection valve using a software programme developed by CapSenze^TM^ (Lund, Sweden). Table 1 gives a summary of the injection sequence and the parameters used.

First, carrier buffer (10 mM phosphate buffer containing 50 mM NaCl, pH 7.4) was run through the system until the working electrode showed a stable baseline. Next, 200 µL of the target analyte (MCLR) was passed into the immunocolumn to specifically bind to the immobilized mAbs, followed by washing off weakly or unbound MCLR molecules using a wash buffer. Then 200 µL HRP-labeled MCLR (tracer) was introduced to bind to the remaining mAbs sites, after which the unbound molecules were again washed off with 400 µL of the same carrier buffer. The catalytic reaction was started with the addition of 200 µL of the ABTS substrate and ABTS^●+^ was produced. The continuously flowing carrier buffer pushed the enzymatic product (ABTS^●+^) from the immunocolumn to the detection flow-cell for amperometric determination using an Autolab PGSTAT12 potentiostat equipped with GPES software (Eco Chemie, Schiedam, The Netherlands).

**Figure 3:**
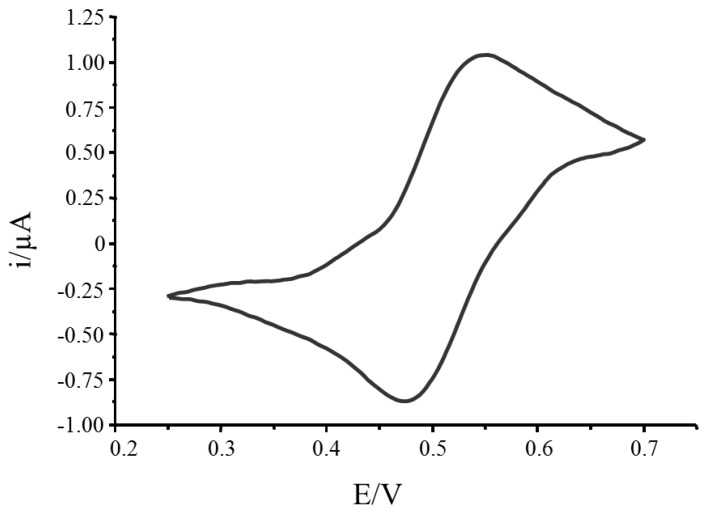
Cyclic voltammogram of Super AquaBlue^TM^ showing both oxidation (anodic) and reduction (cathodic) peaks of ABTS on glassy carbon electrode (vs. Ag/AgCl) with 0.4 V applied potential in Super AquaBlue^TM^.

Amperometric measurements were made in a custom-made electrochemical flow-through cell. The electrode package used in this work contained screen printed electrodes (SPEs) in which the working and auxiliary electrode were made of carbon and the reference electrode was silver. However, it was found that silver reference electrode was non-reusable, consequently allowing only one measurement. To extend the usage of SPEs, an external reference electrode (Ag/AgCl) was therefore introduced to allow multiple measurements. The flow-cell was built to fit the SPEs and Ag/AgCl reference electrode (Figure 2b insert). The amperometric measurement set-up is shown in Figure 2. Upon ABTS oxidation, the catalytic product (ABTS^●+^) was passed into the flow-cell where it gained an electron and becomes reduced, and a transient current signal was recorded. The applied potential for a reduction reaction was determined by performing cyclic voltammetry using the same Autolab PGSTAT12 potentiostat and the appropriate potential was determined by sweeping the potential range from 0.25 to 0.7 V at a scan rate of 0.05 V/s (Figure 3). The column was regenerated for the next assay using a 200 mM glycine-HCl (pH 2.5) solution upon completion of the assay. This was to dissociate both MCLR and MCLR-HRP from the antibodies in the immunocolumn and to remove them.

### 2.5. Selectivity of the Immunocolumn Against Interferences

The influence of the system on non-specific binding was evaluated using possible interfering biomolecules; bovine serum albumin (BSA), aflatoxin B_1_ (AFB_1_) and deoxynivalenol (DON). In place of the MCLR, 250 µL of 1 µg/L of each of the above-mentioned samples were used and the recorded signal responses were compared to that of the carrier buffer (PBS blank) and fixed concentrations of the target analyte (MCLR).

## 3. Results and Discussion

### 3.1. Antibody Immobilization on CNBr-Activated Sepharose Beads

Since strong attachment of the antibody to the support surface is required for online flow and/or sequential injection analysis, mAbs were covalently immobilized on the CNBr-activated Sepharose beads. This was performed mainly to prevent antibodies from being stripped off and lost in the flow, especially if high flow-rates were used. The efficiency of immobilization could not be quantitatively determined since the amount of unbound antibody in the supernatants was too small for the protein determination by available methods. Despite the fact that silver staining is known to detect proteins at a nanogram level [33], our attempts to determine the concentrations of antibodies in the supernatants using SDS-PAGE with silver staining were unsuccessful. Only the band of the starting antibody concentration (0.5 mg/L) was visible (data not shown). Therefore, it was assumed that most of the antibody molecules were successfully attached to the beads.

### 3.2. Amperometric Measurements of ABTS on Glassy Carbon Electrode by Sequential Injection

ABTS is a good electron mediator that exhibits fast and effective electron transfer (kinetics) between the electrode and HRP [34]. In the presence of H_2_O_2_, electrochemical oxidation of ABTS in solution takes place at 0.48 V (vs. Pt) [30,35] to form ABTS^●+^ (cation). In this work, an ABTS substrate (Super AquaBlue^TM^) was used and the oxidation and reduction potentials were studied by cyclic voltammetry. Super AquaBlue^TM^ is a substrate containing ABTS that can be oxidized in the absence of H_2_O_2_ [36]. Eliminating the use of H_2_O_2_ is an advantage for the system since fewer reagents are used.

The oxidation peak produced from ABTS electrochemical reaction was observed at 0.56 V while a reduction peak was shown at 0.48 V (vs. Ag/AgCl) as depicted in Figure 3. According to these results, 0.48 V should be selected as an optimum reduction potential to reduce the ABTS^●+^, enzymatic product from the immunocolumn. However, the electrochemical oxidation process starts at around 0.42 V, way before reaching the peak at 0.56 V. To avoid the electrochemical oxidation of the substrate which could produce extra ABTS^●+^ from excess substrate, 0.4 V was then selected as a working reduction potential instead of 0.48 V. The amount of ABTS^●+^ generated was measured and related to the toxin concentration captured in the column.

**Figure 4:**
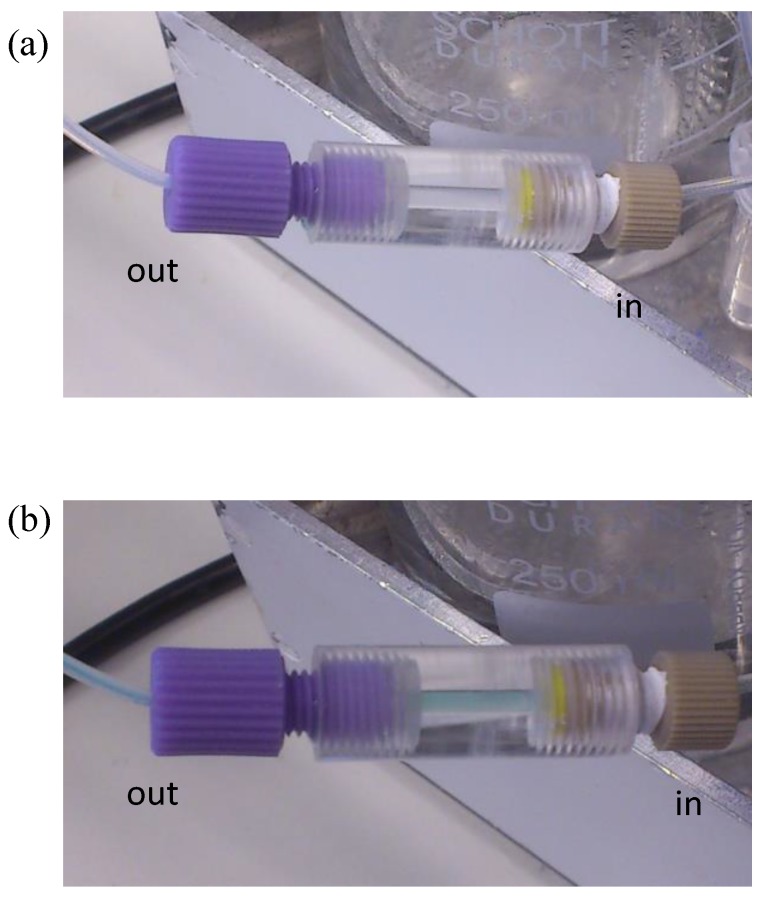
Photographs showing (**a**) clear column before the reaction with substrate and (**b**) blue column during reaction.

Oxidation of ABTS forms a blue/green coloured product [25,35] as observed in Figure 4. Figure 4a shows a clear colour seen before the reaction, and then the blue colour, which could be easily observed inside the immunocolumn (Figure 4b). This colour formation is the result of the biocatalytic reaction of HRP with ABTS forming ABTS^●+^ [25,30]. The colour intensity that relates to the concentration of ABTS^●+^ is inversely proportional to the concentration of the MCs.

**Figure 5:**
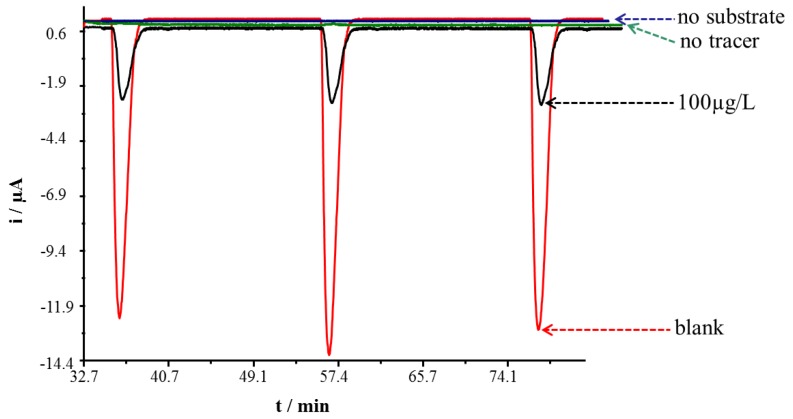
Assay confirmation on different conditions (no substrate, no tracer, 100 µg/L MCLR and PBS (10 mM phosphate buffer containing 50 mM NaCl, pH 7.4) as the blank).

Figure 5 indicates the different responses when the immunocolumn was tested under different conditions for confirmation of enzyme activity. The average B_0_ (maximum signal obtained from blank sample) and highest concentration of MCLR used (100 µg/L) recorded were 11.2 ± 1.4 and 3.5 ± 0.8 µA, respectively. There was no current change when either the substrate or tracer was excluded, suggesting that there was no enzymatic reaction taking place inside the immunocolumn. All the averaged data points were reproducible and repeatable between 3 columns.

**Figure 6:**
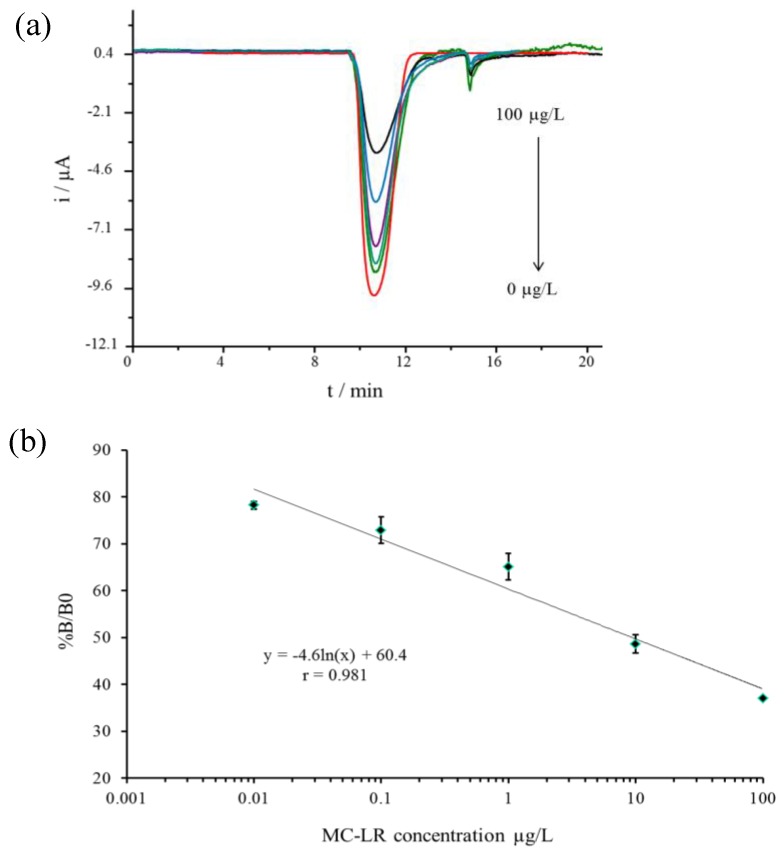
(**a**) Current response of different concentrations of MCLR and blank (0 µg/L); (**b**) standards curve created from concentration between 0.01 and 100 µg/L MCLR.

The electrochemical responses of different MCLR concentrations are presented in Figure 6a. The lowest and highest detection limits were based on 20 and 80% of decrease relative to maximum signal (100%) given by the blank respectively. Figure 6b shows the calibration curve plotted for MCLR concentrations between 0.01 and 100 µg/L. The calibration equation was y = −4.6 ln(x) + 60.4 (r = 0.9863 (Figure 6b). 

The lowest detection limit (0.01 µg/L) is 100 times lower than the acceptable limit for WHO (1 µg/L), however the sensitivity is low when compared to what is achieved with the capacitive measurements using the same mAbs [37].

### 3.3. Selectivity of the Assay

**Figure 7:**
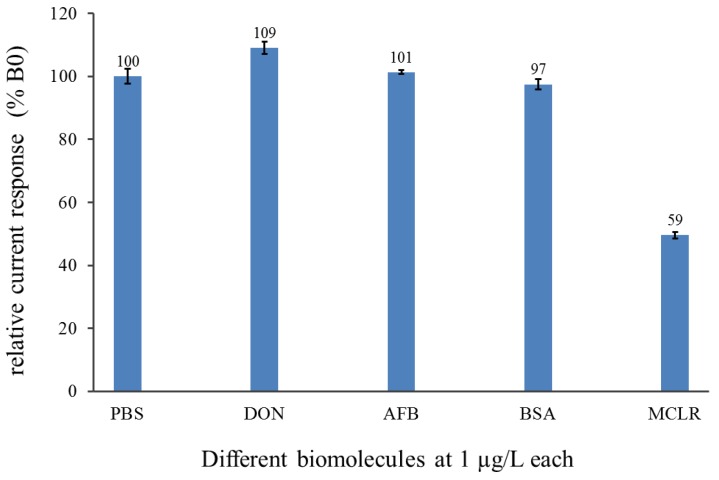
Comparison of 1 µg/L of each of DON, AFB_1_ and BSA against 1 µg/L of MCLR and blank PBS.

The binding specificity of the immunocolumn to MCs was assessed with some possible interfering biomolecules and compared with PBS as blank (Figure 7). Selectivity experiments were performed by diluting unrelated toxins—aflatoxin B1 (AFB_1_) and deoxynivalenol (DON)—and a globular protein, bovine serum albumin (BSA), in buffer to obtain a final concentration of 1 µg/L. Each biomolecule sample was injected into the VersAFlo-system using the same conditions as when MCLR were analyzed. The biomolecule samples resulted in formation of a deep blue product. The maximum amperometric signal was between 97% and 106% relative to 100% of blank buffer. A significant current reduction with MCLR (target) (59%) was observed compared to when only buffer (100%) was present. The pronounced signal reduction observed in the presence of MCs indicates that the immunocolumn specifically interacts with MCs but not with other molecules, which did not cause a reduction in the registered signals. The results further demonstrate that immobilized antibodies do not recognize the tested biomolecules, thereby excluding some false signals that could result from non-specific interactions.

## 4. Conclusions

The VersAFlo-amperometric system is suitable for detection of MCLR down to 0.01 µg/L, which is within the WHO requirement of 1 µg/L of MCLR equivalent in drinking water. The system is also selective towards MCLR and does not recognize potential interferants such as BSA, DON and AFB_1_. Moreover, this system could also be used to detect other MC variant since it uses Adda-specific mAbs. VersAFlo-amperometric detection combines the sensitive amperometric detection with sequential injection of small amount of sample using a simple instrumentation, thereby achieving a more rapid and easy analysis of MCs. One assay cycle takes 20 min with the reaction between substrate and analyte taking less than 3 min. The reaction could be observed in real-time by visualizing the colour change in the column and then by the current response in the electrochemical flow-cell. In addition, with complete automation, flow-rates can be manipulated to achieve desired conditions, as well as minimizing errors that could result from manual sample handling.

Although the sequential flow system presented so far is limited to narrow processing ranges of MCs, it has demonstrated that sequential flow controls can be applied to immunoassays/ELISA to eliminate labour-intensive procedures and long processing times. This type of set-up offers the advantage in that the colour conversion can be observed by the naked eye. However, the reusability of the SPE represents a challenge, as only six measurements could be made with the same electrode without any significant loss in electrochemical detection sensitivity. A further advantage is that the immunocolumn with immobilized antibodies in the flow-system preceding the amperometric detection set up gives the system versatility since a broad spectrum of detectors can be used. In the future, we plan to perfect this system and adapt the assessment of microcystins and related cyanobacterial peptides in real water samples.

## Figures and Tables

**Table 1:** Summary of sequential injection parameters used for one assay cycle.

Reagent	Volume (µL)	Flow Rate (µL/s)	Duration (min)
Sample	200		
Carrier buffer	400	1.67	6
Tracer	200		
Carrier buffer	400	1.67	6
Substrate	200		
Carrier buffer	1000	4.17	5
Regeneration	250		
Carrier buffer	500	4.17	3
